# Enhanced anti-liver tumor efficacy of chimeric antigen receptor-T cells via SATB1 modulation

**DOI:** 10.1038/s41419-025-08307-3

**Published:** 2025-12-10

**Authors:** Lin Zhang, Chenxi Cheng, Xinyi Bi, Jiani Cao, Xiaoyan Li, Tongbiao Zhao

**Affiliations:** 1 State Key Laboratory of Organ Regeneration and Reconstruction, Institute for Stem Cell and Regeneration, Institute of Zoology, Chinese Academy of Sciences, Beijing, China; 2 University of Chinese Academy of Sciences, Beijing, China; 3 Beijing Institute for Stem Cell and Regenerative Medicine, Beijing, China

**Keywords:** Immunization, Cancer

## Abstract

Although Chimeric antigen receptor (CAR) T-cell therapy has achieved remarkable success in treating hematopoietic malignancies, its clinical application in solid tumors is profoundly hindered by persistent T-cell exhaustion within the immunosuppressive tumor microenvironment (TME). Here, we identified SATB1—a genome organizer regulating chromatin architecture—as a key suppressor of CAR-T cell exhaustion. In Glypican-3 (GPC3)-targeted CAR-T cells, SATB1 was significantly downregulated in tumor-infiltrating exhausted populations. SATB1 overexpression not only reduced expression of multiple inhibitory receptors (PD-1, CTLA-4, TIM3, and LAG-3) but also promoted a central memory phenotype, enhancing cytokine production and cytotoxicity against hepatocellular carcinoma (HCC) cells in vitro. In vivo, SATB1-engineered CAR-T cells exhibited superior tumor control and promoted survival, accompanied by reduced exhaustion markers in tumor-infiltrating T cells. These functional improvements are consistent with the reported role of SATB1 in modulating T cell exhaustion, positioning it as a multifunctional enhancer of CAR-T cell fitness. Collectively, our study unveils SATB1 as a multifunctional modulator that simultaneously targets exhaustion and memory differentiation, offering a novel strategy to enhance CAR-T efficacy against solid tumors.

## Introduction

Adoptive cellular immunotherapy (ACT) based on functional immune cell transfer holds great promise in treating various malignant diseases, especially cancer [1, 2]. Among ACT strategies, chimeric antigen receptor (CAR-T) cell therapy has emerged as an effective clinical strategy for the treatment of several hematopoietic malignancies [3–6]. However, its efficacy in solid tumors, including hepatocellular carcinoma (HCC), remains limited due to antigenic heterogeneity, suboptimal infiltration, and immunosuppressive tumor microenvironment (TME)-driven T cell exhaustion[7, 8]. Overcoming these challenges is critical for improving the antitumor efficacy of CAR-T cell therapy for solid tumors.

T cell exhaustion is a state characterized by diminished proliferation, impaired effector function, transcriptional and epigenetic alterations, and upregulation of inhibitory receptors such as Programmed Death-1 (PD-1), Cytotoxic T-Lymphocyte Associated Protein 4 (CTLA-4), and Lymphocyte-Activation Gene 3 (LAG-3) [9–13]. In CAR-T cells, exhaustion is primarily driven by persistent antigenic stimulation and sustained autoactivation due to CAR structure aggregation [12, 14]. Recent efforts to combat T cell exhaustion have focused on modulating transcriptional regulators such as c-Jun [15], NR4A family members [16, 17], BATF (from the AP-1 family) [18, 19], FOXO1 [20, 21], Id2 [22], and Stat5 [23]. Genetic modulation of these factors has been shown to improve CD8^+^ T cell and CAR-T cell function by mitigating exhaustion, enhancing stem-like properties or metabolic adaptability, ultimately leading to tumor regression and extending survival in preclinical models. Moreover, the epigenetic reprogramming underlying T cell exhaustion remains poorly understood, hindering the development of durable solutions.

Special AT-Rich Sequence Binding Protein 1 (SATB1) is a critical genome organizer that reprograms chromatin structure and broadly regulates transcriptional profiles to promote tumor cell proliferation [24] and metastasis [25, 26]. Beyond its oncogenic functions, predominantly expressed in thymocytes, SATB1 is indispensable for thymocyte development [27, 28] and T cell differentiation [29–31]. The absence of SATB1 disrupts thymocyte development, particularly during the double-positive stage [32]. Recent studies have highlighted SATB1’s role in the anti-tumor function of cytotoxic T lymphocytes (CTL) through its recruitment of the nucleosome remodeling deacetylase (NuRD) complex at genomic regions, thereby regulating PD-1 expression and suggesting its potential role in mitigating T cell exhaustion [33]. Additionally, within TME, TGF-β-mediated SATB1 silencing promotes follicular helper T (Tfh) cell differentiation and tertiary lymphoid structures (TLS) formation, further underscoring its multifaceted role in immune regulation and tumor immunity [34].

In this study, we identified SATB1 as a key regulator of T cell exhaustion in Glypican-3 (GPC3)-targeting CAR-T cells within hepatocellular carcinoma (HCC) xenograft models. We observed significant downregulation of SATB1 and concomitant upregulation of PD-1 in tumor-infiltrating exhausted CAR-T cells. We hypothesized that SATB1 overexpression could reprogram CAR-T cell epigenetics to resist exhaustion and enhance anti-tumor efficacy in HCC. Overexpression of SATB1 enhanced the immunophenotypic characteristics, improved effector function, and reduced exhaustion levels in CAR-T cells in vitro. Subsequent studies demonstrated that SATB1-overexpressing CAR-T cells exhibited improved resistance to exhaustion and superior immunotherapeutic efficacy in vivo, leading to accelerated tumor eradication and prolonged survival of tumor-bearing mice. These findings suggest that SATB1 modulation represents a promising strategy to enhance the efficacy of CAR-T cells in treating solid tumors.

## Results

### *SATB1* is downregulated in tumor-infiltrating exhausted T cells

Exhausted T cells in TME are characterized by the upregulation of immune checkpoint molecules like PD-1, reduced proliferative capacity, and impaired effector functions [10, 13, 14]. To better elucidate the transcriptional profiles of tumor-infiltrating exhausted T cells, we analyzed transcriptional profiles across models. In a B78ChOVA melanoma mouse model (GSE201071), *Satb1* mRNA expression was preferentially downregulated in tumor-infiltrated exhausted OT-1 T cells at days 4 and 14 compared to naïve and effector T cells (Fig. S1A) [35]. This finding was corroborated in chronically activated T cells from an LCMV Arm5 infection model (GSE88987), where SATB1 expression decreased in exhausted populations (Fig. S1B) [16].

To assess clinical relevance, we analyzed HCC patient data (GSE111389) and observed consistent SATB1 downregulation in PD-1 high (PD-1 hi) tumor-infiltrating CD8^+^ T cells across all six patients (Fig. S1C) [36]. Further validation using the Pan-Cancer Human T Cell Atlas (scRNA-seq data) revealed high SATB1 expression in naïve and memory T cells, but marked reduction in exhausted subsets (Figs. S1D and S1E) [37]. Collectively, these results implicate SATB1 downregulation as a conserved feature of T cell exhaustion. Engineering CAR-T cells to overexpress SATB1 may represent a promising strategy to mitigate exhaustion and enhance the efficacy of CAR-T cell therapy against solid tumors.

### The potent antitumor activity of GPC3-Targeted CAR-T Cells in Hepatocellular Carcinoma

To investigate the role of SATB1 in exhausted CAR-T cells, we developed a GPC3-targeted CAR-T model. GPC3, a membrane-bound heparan sulfate proteoglycan highly expressed in 70% of HCC patients but absent in normal adult tissues [38], is under clinical evaluation in 11 of 22 ongoing HCC CAR-T trials [39–41]. Our analysis confirmed high GPC3 expression in human HCC cell lines Huh7, HepG2, and Hep3B but not in SK-HEP-1 (Fig. S2A).

Subsequently, second-generation GPC3-CAR-T cells were engineered using an EF1α-promoter lentiviral vector (Fig. S2B) [41]. Primary human T cells, stimulated with anti-CD3/anti-CD28 Dynabeads and IL-2, achieved 60% transduction efficiency (Fig. S2C). These CAR-T cells specifically secreted IFN-γ/IL-2 (Fig. S2D) and lysed GPC3^+^ HCC lines (Huh7, HepG2, Hep3B), but not GPC3^−^ SK-HEP-1 cells (Fig. S2E and S2F), demonstrating their target-dependent specificity and efficacy.

To further evaluate the anti-tumor effects of GPC3-CAR-T cells in vivo, we established a cell line-derived xenograft (CDX) model by subcutaneously injecting GFP/Luc^+^ Huh7 into immunodeficient NCG mice. Following tumor engraftment, mice were intravenously infused with either Ctrl-T or CAR-T cells, and the tumor size was measured at the indicated time points (Fig. S2G). CAR-T cell treatment induced significant tumor regression and improved survival of tumor-bearing mice compared to Ctrl-T cells (Fig. S2H–S2J). However, incomplete regression in some tumors highlighted the need to enhance therapeutic potency—a goal addressed by SATB1 engineering in subsequent studies.

### SATB1 is downregulated in tumor-infiltrating exhausted CAR-T cells

**Fig. 1:**
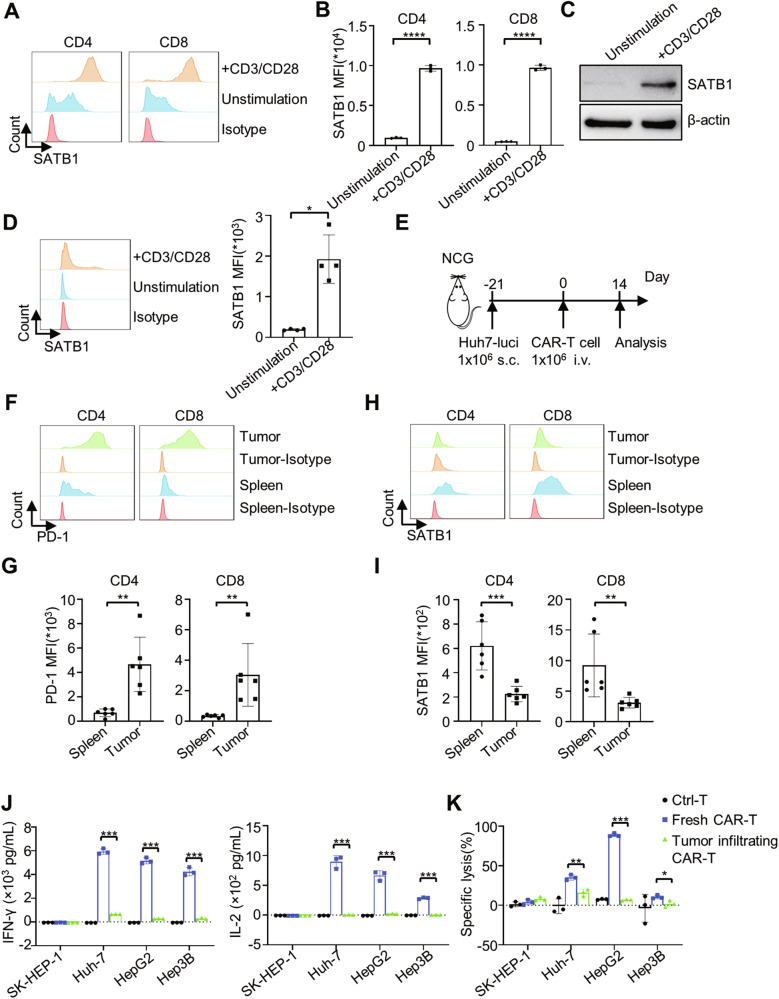
SATB1 is downregulated in tumor-infiltrating CAR-T cells. **A** SATB1 expression in resting and activated human CD4^+^ and CD8^+^ T cells was detected by flow cytometry. **B** Quantification of SATB1 MFI of T cells in (**A**). Data were shown as mean ± SD, *n* = 3; ****, *P* < 0.0001; Student’s *t*-test. **C** Western Blot analysis was performed to detect SATB1 protein levels in resting and activated human T cells. **D** SATB1 expression in resting and activated mouse T cells was measured by flow cytometry. Data were shown as mean ± SD, *n* = 4; *, *P* < 0.05; Mann–Whitney test. **E** Schematic of GPC3-CAR-T cell therapy for HCC CDX model. **F** PD-1 expression on the surface of CD4^+^ and CD8^+^ CAR-T cells isolated from the spleen and tumor was evaluated by flow cytometry. **G** Quantification of PD-1 expression in (**F**). Data are shown as mean ± SD, *n* = 6; **, *P* < 0.01; Student’s *t*-test and Mann-Whitney test. **H** SATB1 expression in spleen and tumor-derived CD4^+^ and CD8^+^ CAR-T cells was detected by flow cytometry. **I** SATB1 MFI quantification in (**H**). Data are shown as mean ± SD, *n* = 6; ***, *P* < 0.001; **, *P* < 0.01; Student’s *t*-test and Mann–Whitney test. **J** IFN-γ and IL-2 secre*t*ion by Ctrl-T, Fresh CAR-T and Tumor infiltrating CAR-T cells co-cultured with HCC tumor cells (3:1 E:T ratio, 18 h) was detected. Data are shown as mean ± SD, *n* = 3; ***, *P* < 0.001; Student’s *t*-test. **K** Cytotoxicity of Ctrl-T, Fresh CAR-T and Tumor infiltra*t*ing CAR-T cells against GFP/Luc^+^ HCC cells was evaluated. Data are shown as mean ± SD, *n* = 3; ***, *P* < 0.001; **, *P* < 0.01; *, *P* < 0.05; Student’s *t*-test.

We next asked whether SATB1 downregulation occurs in CAR-T cells within tumors. We analyzed SATB1 expression in human T cells under resting or anti-CD3/CD28-activated conditions. Consistent with prior studies, SATB1 was highly expressed in activated human CD4^+^ and CD8^+^ T cells (Fig. 1A–C) [33]. Similarly, anti-CD3/CD28 stimulation significantly increased SATB1 levels in mouse splenic CD3^+^ T cells (Fig. 1D).

We transferred GPC3-CAR-T cells into GFP/Luc^+^ Huh7 xenograft-bearing mice to detect SATB1 expression in CAR-T cells within tumors (Fig. 1E). Two weeks post-infusion, tumor-infiltrating CAR-T cells exhibited elevated PD-1 expression (Fig. 1F, G), resembling endogenous exhausted CD8^+^ T cells. Strikingly, SATB1 levels were significantly reduced in these cells compared to splenic CAR-T counterparts, corroborating the RNA-seq data (Fig. 1H, I). Meanwhile, in comparison to fresh CAR-T cells, tumor-infiltrating CAR-T cells exhibited markedly diminished cytokine secretion and cytotoxic activity upon in vitro rechallenge with HCC cell lines (Fig. 1J, K). These findings suggest that SATB1 downregulation is associated with CAR-T cell exhaustion in the TME, highlighting its potential as a target to enhance functionality.

### SATB1 efficiently enhances CAR-T cell functions in vitro

**Fig. 2:**
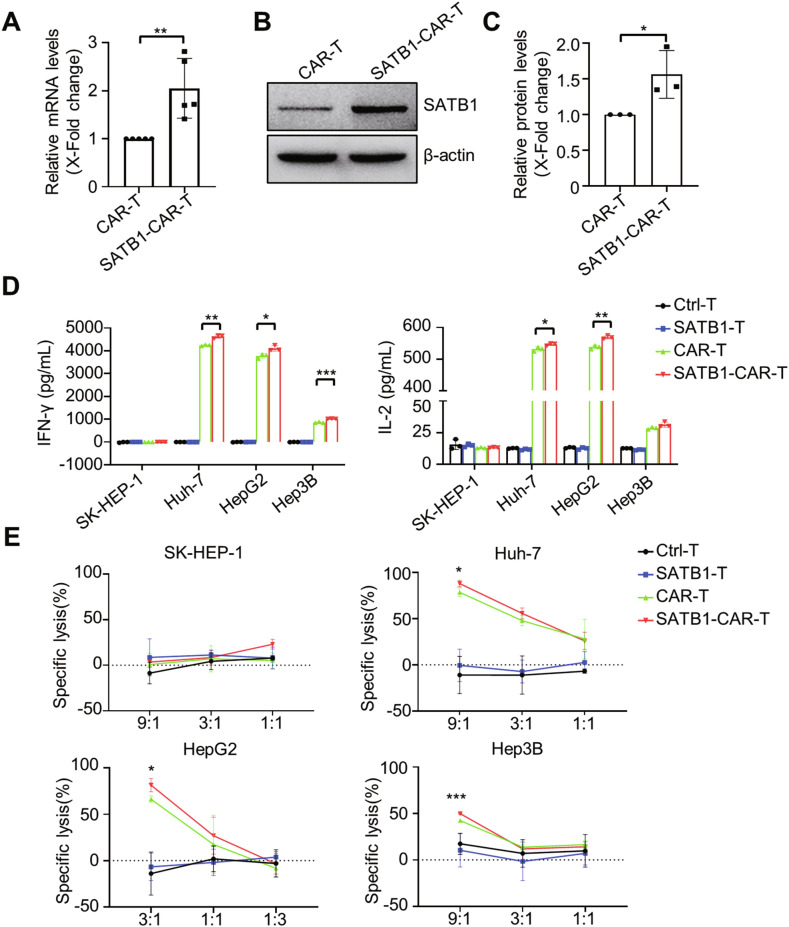
SATB1 enhances the cytotoxicity of CAR-T cells in vitro. **A** Human SATB1 mRNA levels in CAR-T and SATB1-CAR-T cells were quantified by real-time PCR. Data were shown as mean ± SD, *n* = 5; **, *P* < 0.01; Student’s *t*-test. **B** SATB1 protein expression in CAR-T and SATB1-CAR-T cells was assessed by Western blot, with β-actin as the loading control. **C** Relative SATB1 protein levels in (**B**) were quantified and shown as mean ± SD, *n* = 3; *, *P* < 0.05; Student’s *t*-test. **D** IFN-γ and IL-2 secretion by activated CAR-T cells co-cultured with GPC3^+^ HCC tumor cells (1:1 E:T ratio, 18 h) was detected. Data are shown as mean ± SD, *n* = 3; ***, *P* < 0.001; **, *P* < 0.01; *, *P* < 0.05; Student’s *t*-test. **E** Cytotoxicity of Ctrl-T, SATB1-T, CAR-T, and SATB1-CAR-T cells against GFP/Luc^+^ HCC cells was evaluated. Data are shown as mean ± SD, *n* = 3; ***, *P* < 0.001; *, *P* < 0.05; Student’s *t*-test.

Considering the downregulation of SATB1 in tumor-infiltrated exhausted CAR-T cells, we investigated whether SATB1 overexpression could enhance CAR-T cell functionality. We co-transduced human T cells with either an empty vector or a SATB1 overexpression vector alongside the CAR construct. Successful SATB1 overexpression in SATB1-CAR-T cells was confirmed at both the mRNA (Fig. 2A) and protein levels (Fig. 2B, C).

To evaluate the functional impact of SATB1 overexpression, Ctrl-T, SATB1-overexpressing T (SATB1-T), CAR-T, or SATB1-CAR-T cells were co-cultured with HCC cell lines (SK-HEP-1, Huh-7, HepG2, and Hep3B). SATB1-CAR-T cells exhibited enhanced cytokine release compared to conventional CAR-T cells (Fig. 2D). Consistently, SATB1-CAR-T cells exhibited increased killing efficiency against GFP/Luc^+^ Huh-7, HepG2, and Hep3B cells (Fig. 2E). These results indicate that SATB1 could enhance the anti-tumor efficacy of CAR-T cells in vitro, highlighting its potential as a strategy to improve the antitumor efficacy of CAR-T cells.

### SATB1 modulates the immunophenotypes of CAR-T cells in vitro

**Fig. 3:**
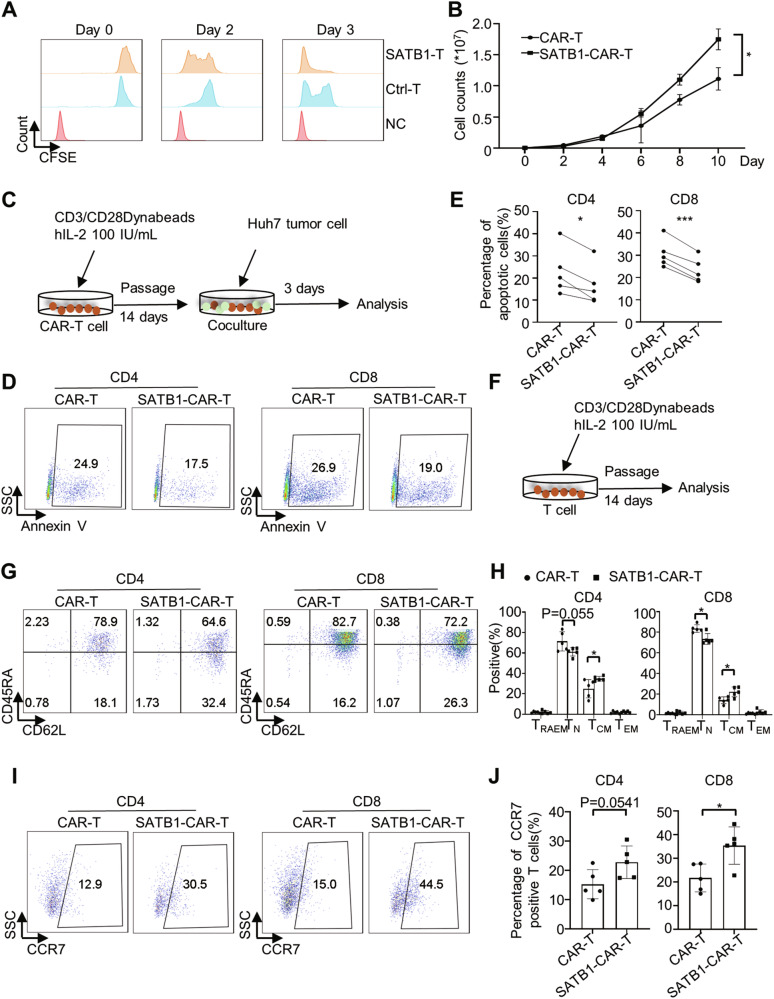
Overexpression of SATB1 affects CAR-T cell immunophenotypes in vitro. **A** CFSE stained Ctrl-T and SATB1-T cells were cultured for 3 days, and proliferation was analyzed by flow cytometry. **B** SATB1 overexpression promoted CAR-T cell proliferation in vitro by cell counting analysis. Data were shown as mean ± SD, *n* = 3; *, *P* < 0.05; Student’s *t*-test. **C** Schematic of CAR-T cell apoptosis levels detection by incubation with Huh7 tumor cells. **D** SATB1-CAR-T cells exhibited lower apoptosis levels compared to CAR-T cells after incubation with Huh7 tumor cells (1:1 E:T ratio, 3 days). **E** Quantification of Annexin V-positive T cells (shown in **D**). Data are shown as mean ± SD, *n* = 5; ***, *P* < 0.001; *, *P* < 0.05; paired two-tailed *t*-test. **F** Schematic of CAR-T cell subsets detection. **G** CD45RA and CD62L expression in CAR-T and SATB1-CAR-T cells were analyzed by flow cytometry. **H** Percentages of T cell subtypes from (**G**) were shown as mean ± SD, *n* = 5; *, *P* < 0.05; Student’s *t*-test and Mann–Whitney test. **I** CCR7 expression in CAR-T and SATB1-CAR-T cells was analyzed by flow cytometry. **J** Statistics of CCR7-positive T cells in (**I**). Data are shown as mean ± SD, *n* = 5; *, *P* < 0.05; Student’s *t*-test.

Given the enhanced functionality of SATB1-CAR-T cells, we investigated the influence of SATB1 overexpression on T cell biological properties and immunophenotypic profiles. SATB1-T and SATB1-CAR-T cells exhibited significantly enhanced proliferation over a two-week culture period compared to controls (Fig. 3A, B). Moreover, after co-culture with GPC3^+^ Huh7 cells for 3 days (Fig. 3C), SATB1-CAR-T cells exhibited reduced apoptosis levels, indicating improved resistance to tumor-induced stress (Fig. 3D, E).

To explore the underlying phenotypic changes after in vitro culture for 14 days (Fig. 3F), we analyzed T cell differentiation markers CD45RA and CD62L, which classify primary human T cells into four kinds of differentiation subsets: CD45RA^+^ CD62L^+^ naïve T cells (T_N_), CD45RA^−^ CD62L^+^ central memory T cells (T_CM_), CD45RA^−^ CD62L^−^ effector memory T cells (T_EM_), and CD45RA^+^ CD62L^−^ effector memory T cells (T_RAEM_) [42, 43]. Strikingly, SATB1 overexpression increased CD45RA^−^ CD62L^+^ central memory T cells (T_CM_) subset both in CD4^+^ and CD8^+^ T cells (Figs. 3G, H and S3A–S3B). This memory reprogramming was further supported by elevated CCR7 expression, a key mediator of T cell homeostasis, lymphoid homing, and sustained anti-tumor responses (Figs. 3I, J and S3C, S3D).

Notably, SATB1 overexpression had no effect on CD4^+^ or CD8^+^ T cell subset distribution (Fig. S3E) and did not alter regulatory T cell (T_reg_) proportions (Fig. S3F), suggesting selective modulation of effector/memory subsets without perturbing other immune cells.

### SATB1 attenuates TGF-β-induced Immunosuppression in T Cells

SATB1 has been shown to recruit the nucleosome remodeling deacetylase (NuRD) complex to *Pdcd1* regulatory regions, and the loss of *Satb1* increases PD-1 expression upon T cell activation [33]. Consistent with the mechanism, after in vitro culture for 14 days (Fig. S4A), SATB1-T cells exhibited significantly reduced PD-1 expression compared to controls (Fig. S4B), while other exhaustion markers (CTLA-4, TIM3, LAG-3) remained unchanged (Fig. S4C).

Transforming growth factor-beta (TGF-β), mainly secreted by immunosuppressive cells and tumor cells, limits CAR-T cell efficacy in solid tumors by suppressing T cell activation, proliferation, migration, and differentiation [44–46]. Therapeutic strategies targeting TGF-β signaling have shown considerable promise in preclinical and clinical studies [47–49]. In line with previous research, after TGF-β1 treatment (Fig. S4D), we detected downregulated SATB1 expression and increased PD-1 levels in Ctrl-T cells (Fig. S4E–S4H). Strikingly, SATB1-T cells resisted this regulation, maintaining significantly higher SATB1 and lower PD-1 expression compared to Ctrl-T cells under TGF-β1 exposure (Fig. S4E–S4H). These findings suggest that SATB1 overexpression may counteract TGF-β-driven exhaustion, potentially preserving T cell functionality in immunosuppressive microenvironments.

### SATB1 ameliorates CAR-T cell exhaustion in vitro

To systematically evaluate CAR-T cell exhaustion in vitro, we established a repetitive co-culture model with Huh7 tumor cells (Fig. S5A). After three rounds of co-incubation, conventional CAR-T cells showed significant upregulation of exhaustion-associated markers, including PD-1, CTLA-4, TIM3, and LAG-3, confirming successful exhaustion induction (Fig. S5B–S5E).

Consistent with the suppressive effect of SATB1 on PD-1 under TGF-β exposure (Fig. S4), SATB1 overexpression markedly reduced PD-1 expression in both CD4^+^ and CD8^+^ CAR-T cells post-co-culture (Fig. S5F). Furthermore, SATB1-CAR-T cells also showed attenuated upregulation of CTLA-4, TIM3, and LAG-3 (Fig. S5G–S5I). These data collectively suggest that SATB1 overexpression could ameliorate CAR-T cell exhaustion in vitro, potentially preserving their anti-tumor functionality through epigenetic regulation of immune checkpoint molecules.

### SATB1 enhances immunotherapeutic efficacy of CAR-T cells in vivo

**Fig. 4:**
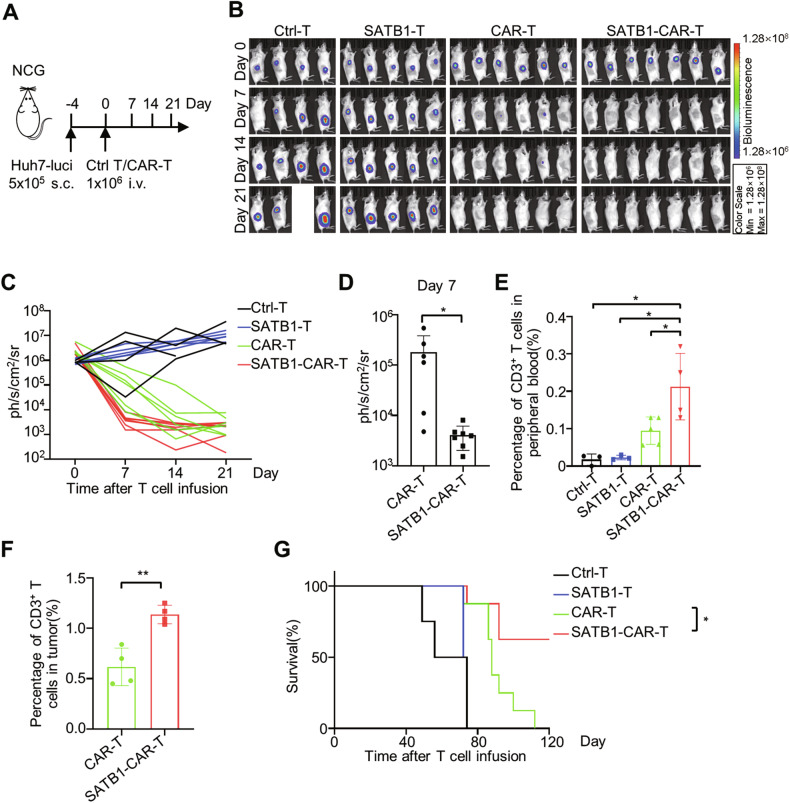
Overexpression of SATB1 enhances the efficiency of CAR-T cells in vivo. **A** Schematic of the human liver cancer CDX model. **B** Tumor luminescence signals after infusion of Ctrl-T, SATB1-T, CAR-T and SATB1-CAR-T cells in the CDX model. **C** Quantification of tumor bioluminescence signals at indicated time points from (**B**). Ctrl-T: *n* = 4; SATB1-T: *n* = 5; CAR-T: *n* = 6; SATB1-CAR-T: *n* = 7. **D** Statistics of tumor bioluminescence signals on day 7 after CAR-T and SATB1-CAR-T cells treatment. Data are shown as mean ± SD, CAR-T: *n* = 6; SATB1-CAR-T: *n* = 7; *, *P* < 0.05; Student’s *t*-test. **E** Survival of peripheral blood human T cells on day 7 after T cell treatment. Data are shown as mean ± SD, Ctrl-T: *n* = 3; SATB1-T: *n* = 3; CAR-T: *n* = 5; SATB1-CAR-T: *n* = 4; *, *P* < 0.05; Student’s *t*-test. **F** Survival of tumor-infiltrated human T cells after T cell treatment. Data are shown as mean ± SD, CAR-T: *n* = 4; SATB1-CAR-T: *n* = 4; **, *P* < 0.01; Student’s *t*-test. **G** SATB1-CAR-T cell trea*t*ment prolonged the survival of CDX model mice. Ctrl-T: *n* = 4; SATB1-T: *n* = 4; CAR-T: *n* = 8; SATB1-CAR-T: *n* = 8; *, *P* < 0.05; log-rank test.

To assess the in vivo efficacy of SATB1-CAR-T cells, we established a human liver cancer cell line-derived xenograft (CDX) model (Fig. 4A). Mice receiving SATB1-CAR-T cells showed superior tumor regression compared to those receiving conventional CAR-T cells (Fig. 4B, C). Bioluminescence imaging on day 7 confirmed noticeably reduced tumor burden in SATB1-CAR-T-treated mice (Fig. 4B–D).

Flow cytometry analysis revealed nearly 2-fold higher infiltration of human CD3^+^ T cells in both peripheral blood and tumor tissue of SATB1-CAR-T-treated mice (Fig. 4E, F). Notably, tumor-infiltrating CD8^+^ T cells from SATB1-CAR-T group exhibited 50% lower PD-1, CTLA-4, TIM3, and LAG-3 expression compared to controls (Fig. S6), aligning with their exhaustion-resistant phenotype in vitro.

Importantly, SATB1-CAR-T cell therapy significantly extended the survival of tumor-bearing mice, with over 50% surviving beyond 100 days post-infusion (Fig. 4G). Collectively, these findings highlight that SATB1 overexpression enhances CAR-T cell persistence and functionality in solid tumors.

## Discussion

The clinical application of CAR-T cells has rapidly expanded, with 23% of 517 registered clinical trials in China focusing on solid tumors, particularly HCC [4, 8, 39, 50]. Bioinformatics analyses have revealed a TME enriched with exhausted CD8^+^ T cells and regulatory CD4^+^ T_regs_ in HCC, emphasizing the need for engineering exhaustion-resistant CAR-T cells to improve clinical efficacy [37, 51]. Comparative analysis of the unique transcriptional programs, epigenetic programs, and metabolic properties of exhausted T cells has identified several T cell exhaustion-related proteins and transcription factors, such as CD38 [52], Rgs1 [53, 54], and Tigit [55]. While previous studies have focused on highly expressed genes in exhausted T cells, we identified special AT-rich binding protein SATB1, an epigenetic remodeling factor downregulated in exhausted T cells, as a crucial regulator of CAR-T cell exhaustion. Our study demonstrates that SATB1 overexpression mitigates CAR-T cell exhaustion and improves anti-tumor efficacy, potentially not only through mechanisms involving broadly suppression of inhibitory receptors (PD-1, CTLA-4, TIM3 and LAG-3) (Figs. S5 and S6) but also promotion of CD45RA^−^ CD62L^+^ T cell subsets and CCR7^+^ T cell subsets (Figs. 3G–J and S3A–S3D) and enhanced resistance to TGF-β-mediated immunosuppression (Fig. S4) in the HCC microenvironment.

To further explore T cell exhaustion mechanisms, we developed an ex vivo co-culture system for chronic antigen stimulation and rapid T cell exhaustion induction (Fig. S5A) [10, 12, 56, 57]. In our co-culture system, CAR-T cells exhibited significant upregulation of inhibitory receptors (Fig. S5B–S5E). This system, which can be optimized with TGF-β or immunosuppressive cells, provides a platform for screening genes and drugs that enhance CAR-T cell function.

SATB1, initially detected at high levels in thymocytes, progenitor cells (such as osteoblasts), and the epidermal basal layer, plays critical roles in embryogenesis, neurogenesis, and malignancies [58]. It critically regulates hematopoietic development, thymus maturation, and T cell differentiation [27–31]. In mature T cells, SATB1 is dynamically regulated by T cell receptor (TCR) signaling and maintains the distinct naive chromatin state within naive CD8^+^ T cells, also regulates chemokine genes through enhancer-promoter interactions [28, 59–61]. SATB1 also contributes to T cell 3D genome homeostasis and immune tolerance [31, 62, 63]. Recent studies identified SATB1 as an epigenetic negative regulator of PD-1, mitigating T cell exhaustion [33]. Align with its reported role in epigenetic regulation, SATB1 modulation broadly enhance CAR-T cell fitness, addressing both exhaustion and persistence—a dual challenge that single-pathway interventions may incompletely resolve. Future studies are required to directly link SATB1 overexpression to chromatin remodeling in CAR-T cells. Our study further demonstrates that SATB1 overexpression enhances CAR-T cell functionality without altering T_reg_ proportions (Fig. S3F), consistent with its role in maintaining T cell homeostasis [64]. Compared to previous work, the expansion of the T_CM_ subset in SATB1-CAR-T cells supports the superior efficacy of T_CM_ in CAR-T therapy, characterized by robust proliferation capacity, prolonged persistence, and reduced exhaustion [19]. Since SATB1 deletion causes mitochondrial function impairment and oxidative stress in CD4^+^ T cells, its overexpression may preserve mitochondrial integrity in TME, thereby sustaining CAR-T cell functionality [65]. Furthermore, the precise mechanisms by which SATB1 modulates CAR-T-cell immunophenotypes and the potential of SATB1 to enhance CAR-T cell function in other solid tumors warrants further investigation.

In conclusion, our study not only identifies SATB1 as a potential therapeutic target to alleviate CAR-T cell exhaustion but also provides a novel strategy to enhance CAR-T cell efficacy in solid tumors, particularly HCC. While SATB1 overexpression shows promise, potential side effects such as metabolic stress, autoimmune reactions, and cytokine release syndrome (CRS) require further evaluation. These risks could be mitigated through inducible expression systems and close monitoring of cytokine levels. The development of potent small-molecule drugs targeting SATB1 and integration with immune checkpoint inhibitors or multi-targeted CAR-T cells may further improve therapeutic outcomes.

## Materials and methods

### Cell lines and culture conditions

Hepatocellular carcinoma (HCC) cell lines Huh7, HepG2 (HB-8065, ATCC), Hep3B (BNCC360312, BNCC), SK-HEP-1 (HTB-52, ATCC) and Lentiviral producer cell line 293 T (CRL-11268, ATCC) were cultured in Dulbecco’s modified Eagle’s medium (DMEM) (Gibco) supplemented with 10% heat-inactivated fetal bovine serum (FBS) (Vistech), 2 mM GlutaMAX^TM^-I (Gibco), 1 mM sodium pyruvate (Gibco), 0.1 mM nonessential amino acids (Gibco), 100 µg/mL streptomycin and 100 U/mL penicillin (Gibco).

For luciferase-based experiments, the lentivirus of green fluorescent protein and firefly luciferase fusion protein (GFP/Luc) was transduced into tumor cells to produce stable cell lines Huh7-GFP/Luc, HepG2-GFP/Luc, Hep3B-GFP/Luc, and SK-HEP-1-GFP/Luc.

### Animals

The use of animals for this study was approved by the Institutional Animal Care and Use Committee (IOZ2016004). 6–12-week-old C57BL/6J mice were purchased from SPF (Beijing) Biotechnology Co., Ltd. and were used for splenic CD3^+^ T cell activation. NCG (NOD/ShiLtJGpt-*Prkdc* ^em26Cd52^*Il2rg* ^em26Cd22^/Gpt) mice aged 6–12 weeks (Gempharmatech Co., Ltd) were used for human tumor cell line CDX models.

### Generation of CAR constructs

To target GPC3, the chimeric antigen receptor (CAR) was designed based on the single-chain variable fragment (scFv) derived from GC33 antibody, which was linked to the CD8 hinge and transmembrane domain, followed by the intracellular domains of 4-1BB and the signaling moiety of the CD3ζ chain. The CAR sequence was synthesized at Sangon Technology (Shanghai, China) and cloned into the pFUW-EF1α-P2A-eGFP lentiviral vector. CAR-T cells were identified by eGFP expression.

### Lentivirus preparation

The human SATB1 full sequence was synthesized at GenScript Technology (Shanghai, China) and cloned into the pFUW-EF1α-P2A-mCherry lentiviral backbone to generate gene expression plasmid pFUW-EF1α-SATB1-P2A-mCherry. For CAR and gene vector, the pFUW vectors harboring P2A-eGFP and P2A-mCherry sequences were, respectively, used as a negative control. We obtained pLenti-CMV-eGFP-linker-Luc-PGK-Puro lentiviral vector (GFP/Luc) from OBiO Technology (Shanghai, China).

We obtained Lentiviral particles from 293T-packaging cells by calcium phosphate transfection. The CAR or gene vector plasmid, pMD2.G plasmid, and psPAX2 plasmid were transfected into 293T-packaging cells and incubated at 37 °C for 12 h. Then 293T-packaging cells were cultured in fresh medium for 48 h. The supernatants were collected and filtered through 0.45 μm filter. Lentiviral particles were concentrated by ultracentrifugation at 20,000 rpm for 2 h at 4 °C. The viral granules were resuspended in X-VIVO^TM^ 15 medium and filtered through 0.22 μm filter and stored at −80 °C.

### CAR-T cell generation and cell culture

For CAR-T cell generation, human peripheral blood mononuclear cells (PBMCs) were obtained from the Biobank of Peking Union Medical College Hospital through Ficoll-Paque PLUS gradient centrifugation (17-1440-02, GE Healthcare). Primary human T cells were negatively selected from PBMCs with a Pan T Cell Isolation Kit (130-096-535, Miltenyi) and were stimulated CD3/CD28 Dynabeads (11161D, ThermoFisher) at the ratio of 1:1. The T cell culture medium was X-VIVO^TM^ 15 medium (04-418Q, Lonza) supplemented with 5% heat-inactivated FBS (Vistech), 1 mM sodium pyruvate (Gibco), 2 mM GlutaMAX^TM^-I (Gibco), and 100 IU/mL recombinant human IL-2 (200-02, PeproTech). T cells were transduced with control vector, GPC3-CAR, and/or SATB1 overexpression lentiviral particles after 24 h activation and were cultured at a concentration of 10^6^ cells/mL in 6-well plates for 2–3 weeks.

### Quantitative real-time PCR

Total RNA was extracted from sorted mCherry^+^ CAR-T cells and SATB1-CAR-T cells with an RNAeasy Mini Kit (Qiagen). Total RNA (1 µg) was reverse transcribed into cDNA using a StarScript III All-in-one RT Mix with gDNA Remover (A230-10, GenStar). Quantitative real-time PCR was performed with GoTaq® qPCR Master Mix (Promega) and a QuantStudio^TM^ 6 Flex Real-Time PCR System. All samples were analyzed in duplicate and normalized to *GAPDH*. The following primers were used: *GAPDH*-F 5′-GGAGCGAGATCCCTCCAAAAT-3′, *GAPDH*-R 5′-GGCTGTTGTCATACTTCTCATGG-3′, *SATB1*-F 5′-GATCATTTGAACGAGGCAACTCA-3′, *SATB1*-R 5′-TGGACCCTTCGGATCACTCA-3. The threshold cycle was determined, and the relative gene expression ratio was calculated as follows: fold-change = 2^−ΔΔCt^.

### Western blotting and antibodies

Whole-cell lysates of T cells were generated by lysing on ice in RIPA buffer for 30 min containing a protease inhibitor cocktail (04693116001, Roche) and 1 mM PMSF (ST506, Beyotime). Equivalent protein quantities (15 µg) of total protein were run in 10% SDS–PAGE gel (Bio-Rad) and transferred to nitrocellulose membranes (Millipore). The membranes were then blocked with 5% non-fat milk in 1× TBST (T1082, Solarbio) and probed with a primary antibody directed against SATB1 (1:1000, ab109122, Abcam) and β-Actin (1:2500, A5441, Sigma-Aldrich) overnight at 4 °C. After incubation with appropriate HRP-conjugated secondary antibody (Beyotime), signals from bound antibodies were detected with a Luminata Forte Western HRP Substrate Kit (WBLUF0100, Millipore) and quantified using Image J Software.

### Flow cytometry analysis and antibodies

Flow cytometry was used to detect the expression of cell surface markers. The CAR expression was detected by eGFP, and the SATB1 expression was detected by mCherry. CAR-T cells were stained with Human TruStain FcX™ Antibody (422301), Pacific Blue™ anti-human CD45 (368539), APC anti-human CD3 (300411), Brilliant Violet 421™ anti-human CD4 (357423), APC/Cyanine7 anti-human CD8a (301016), APC anti-human CD45RA (304111), FITC anti-human CD62L (980702), PE anti-human CD62L (304805), Alexa Fluor® 647 anti-human CD279 (PD-1) (329910), APC anti-human CD152 (CTLA-4) (369611), APC anti-human CD366 (TIM3) (364803), Brilliant Violet 421™ anti-human CD223 (LAG-3) (369313) and Alexa Fluor® 647 anti-human FOXP3 (320113) antibodies from Biolegend; and Recombinant APC Anti-Glypican-3 (ab275695), Recombinant Anti-SATB1 (ab109122) antibodies from Abcam; and Goat anti-Rabbit IgG (H + L) Cross-Adsorbed Secondary Antibody, Alexa Fluor™ 555 (A-21428) antibody from Invitrogen.

For surface markers detection, T cells were collected, washed with PBS. 1 × 10^6 ^T cells were suspended in 100 µL PBS with 1 µL TruStain FcX™ Antibody for 20 minutes at 4 °C, then incubated with specific surface antibodies for 30 min at 4 °C. For intracellular staining, T cells were stained with surface markers first, then were stained with intracellular antibodies by Foxp3/Transcription Factor Staining Buffer Set (00-5523-00, eBioscience) on the basis of the manufacturer’s instructions. The experiment data were acquired using BD FACS AriaIII and were then analyzed using FlowJo software.

### Apoptosis and proliferation assays

For apoptosis level detection of T cells, we used Annexin V-APC (40310ES20, Yeasen) to stain T cells based on the manufacturer’s instructions after T cell membrane protein staining. To test the proliferation level of T cells, the fluorescent dye CFDA SE was used according to the manufacturer’s instructions. For the cell number counting experiment, T cells (5 × 10^4^) were seeded in a 48-well plate on day 0 and were passaged at a concentration of 10^6^ cells/mL, counted, and recorded every two days.

### Cytotoxicity assay

We evaluated the cytotoxicity of CAR-T cells by co-cultured with luciferase labeled tumor cells in vitro. One day before the experiment, the anti-CD3/CD28 magnetic beads were removed. The target tumor cells (Huh7-GFP/Luc, HepG2-GFP/Luc, Hep3B-GFP/Luc, and SK-HEP-1-GFP/Luc) were seeded in white 96-well plates at a density of 5000 cells/50 µL each well. At a ratio of 9:1, 3:1, 1:1, or 1:3, the indicated T cells were seeded with target tumor cells for 18 h. Target tumor cells alone were determined as the maximal luciferase activity. Then D-luciferin, sodium salt (40901ES03, Yeasen) was prepared and added to each well according to the manufacturer’s instructions. The luminescence signal of each well was measured by a PerkinElmer Victor X3 Reader. The specific lysis rate was calculated using the formula: [(Maximal luciferase activity – Experimental Luciferase Activity)/Maximal luciferase activity] × 100.

### Enzyme-linked immunosorbent assay (ELISA) assay

To analyze the cytokine secretion by CAR-T cells, one day before the experiment, the anti-CD3/CD28 magnetic beads were removed. CAR-T cells and target tumor cells were seeded at a density of 5000 cells/50 µL each well at an effector-to-target ratio of 1:1 in 96-well round-bottom plates (Nunc) for 24 h. The supernatants were collected and cytokines IL-2 and interferon (IFN-γ) were measured using ELISA Kits (70-EK102, 70-EK180, Multi-Science) following the manufacturer’s protocol by ELISA plate reader (EL-808, Biotek). The cytokine concentration was quantified by a standard curve.

### In vivo antitumor model

To assess the antitumor activity of CAR-T and SATB1-CAR-T cells in vivo, we constructed an HCC cell line-derived CDX model. 5 × 10^5^, using at least three mice per group for all in vivo studies to ensure statistical power. Huh7-GFP/Luc cells were subcutaneously (s.c.) injected into NCG mice on the right flank. 4 days after tumor cell implantation, tumor-bearing mice with equal tumor burden were selected and randomized to different treatment groups, and 1 × 10^6^ Ctrl-T, SATB1-T, CAR-T, or SATB1-CAR-T cells were injected intravenously (i.v.) via the tail vein. Tumor-bearing mice were then intraperitoneally (i.p.) injected with 150 mg/kg D-luciferin at indicated time points in a blinded fashion, and luminescence signals were monitored and counted by an IVIS Spectrum Imaging platform (Caliper, Boston, MA, USA). To assess the survival curves, GraphPad Prism Software was used to record and analyze.

### Statistical analysis

All data presented graphically as the mean ± standard deviation (S.D.) was from at least three independent experiments. Each exact *n* value is stated in the corresponding figure legend. We conducted Shapiro-Wilk tests for all statistical data and conducted Student’s *t*-tests for datasets passed normality test. For datasets with significant deviations from normality (p < 0.05), we conducted with Mann–Whitney test (non-parametric alternative) for independent groups and Wilcoxon signed-rank tests for paired data. The log-rank test was performed for comparison of survival curves. All statistical data were analyzed by GraphPad Prism and statistical significance was defined as *P < 0.05, **P < 0.01, ***P < 0.001, and ****P < 0.0001.

## Supplementary information

Supplemental Figure legends

Supplementary Figure S1

Supplementary Figure S2

Supplementary Figure S3

Supplementary Figure S4

Supplementary Figure S5

Supplementary Figure S6

Original Data Files

## Data Availability

The data and materials during the current study are available from the corresponding author upon reasonable request.
