# Supplementary material for: Enhanced anti-liver tumor efficacy of chimeric antigen receptor-T cells via SATB1 modulation
Source: Cell Death Dis. 2025 Dec 10;17(1):93. doi: 10.1038/s41419-025-08307-3 (PMC12830387; doi:10.1038/s41419-025-08307-3)
Supplement: Supplementary file 1 — Supplemental Figure legends [file 41419_2025_8307_MOESM1_ESM.docx]

**Supplementary Figure 1.** Bioinformatic analysis showed that *SATB1* is downregulated in tumor-infiltrating exhausted T cells. (**A**) Heatmap of differentially expressed genes in naive T cells, effector T cells, B78ChOVA melanoma-infiltrating early (D4ex) or late (D14ex) exhausted T cells. (**B**) Heatmap showing differentially expressed genes in P14 naive T cells, effector T cells and exhausted T cells from LCMV Arm5 lentivirus-infected mice. (**C**) Heatmap showing differentially expressed genes in PD-1 high (PD-1 hi) and PD-1 intermediate (PD-1 int) tumor-infiltrating T cells from six liver cancer patients. (**D**) Expression and distribution of SATB1 and PD-1 in tumor-infiltrating CD4^+^ T cell subsets from human pan-cancer. (**E**) Expression and distribution of SATB1 and PD-1 in tumor-infiltrating CD8^+^ T cell subsets from human pan-cancer.

**Supplementary Figure 2.** Establishment of CAR-T cells targeting hepatocellular carcinoma antigen GPC3. (**A**) GPC3 expression on human HCC cell lines SK-HEP-1, Huh7, HepG2 and Hep3B was detected by flow cytometry. (**B**) Schematic of the FUW-EF1α-GPC3CAR-P2A-eGFP vector. (**C**) The transfection efficiency of the GPC3-CAR lentiviral vector. (**D**) GPC3-CAR-T cells secrete high levels of IFN-γ and IL-2 when co-cultured with GPC3-positive HCC tumor cells. Data are shown as mean ± SD, *n*=3; ***, *P* < 0.001; Student’s *t*-test. (**E**) Construction of GFP/Luc^+^ HCC tumor cell lines. (**F**) Cytotoxicity of Ctrl-T and CAR-T cells against GFP/Luc^+^ HCC tumor cell lines. Data are shown as mean ± SD, *n*=3; ***, *P* < 0.001; Student’s *t*-test. (**G**) Schematic of GPC3-CAR-T cells treating GFP/Luc^+^ Huh7-derived CDX model. (**H**) Bioluminescence images of tumor in CDX model after treatment of Ctrl-T or GPC3-CAR-T cells at the indicated time points. (**I**) The bioluminescence signal from (**H**) was recorded at the indicated time points. (**J**) The survival curves for GFP/Luc^+^ Huh7-derived CDX mice receiving Ctrl-T or GPC3-CAR-T cells. Ctrl-T: *n*=6; CAR-T: *n*=6; *, *P* < 0.05; log-rank test.

**Supplementary Figure 3.** SATB1 overexpression did not affect the distribution of T cell subsets. (**A**) CD45RA and CD62L expression in Ctrl-T and SATB1-T cells were analyzed by flow cytometry. (**B**) Percentages of T cell subtypes from (**A**) were shown as mean ± SD, *n*=3; *, *P* < 0.05; Student’s *t*-test. (**C**) CCR7 expression in Ctrl-T and SATB1-T cells was analyzed by flow cytometry. (**D**) Statistics of CCR7-positive T cells in (**C**). Data are shown as mean ± SD, *n*=3; *, *P* < 0.05; Student’s *t*-test. (**E**) SATB1 overexpression does not affect the subpopulations of CD4^+^ and CD8^+^ CAR-T cells. (**F**) Flow cytometry was used to detect FOXP3 expression on CD4^+^ CAR-T and SATB1-CAR-T cells. Data were shown as mean ± SD, *n*=3; ns, no significant difference; Student’s *t*-test.

**Supplementary Figure 4.** SATB1 mitigated the immunosuppression induced by TGF-β treatment. (**A**) Schematic of CAR-T cell background exhaustion level detection. (**B**) Background PD-1 expression on Ctrl-T and SATB1-T cells was detected by flow cytometry. Data are shown as mean ± SD, *n*=5; *, *P* < 0.05; paired two-tailed *t*-test. (**C**) Background CTLA-4, TIM3 and LAG-3 expression on Ctrl-T and SATB1-T cells was detected by flow cytometry. Data are shown as mean ± SD, *n*=5; ns, no significant difference; paired two-tailed *t*-test and Wilcoxon matched-pairs signed rank test. (**D**) Schematic of TGF-β1 treatment during T cell culture *in vitro*. (**E**) SATB1 expression in Ctrl-T and SATB1-T cells after treatment with or without TGF-β1 (5 ng/mL) for 48 h. (**F**) Quantifications of relative SATB1 MFI were shown as mean ± SD, *n*=4; ***, *P* < 0.001; **, *P* < 0.01; *, *P* < 0.05; Student’s *t*-test. (**G**) PD-1 expression in Ctrl-T and SATB1-T cells after treatment with or without TGF-β1 (5 ng/mL) for 48 h. (**H**) Quantifications of PD-1 MFI were shown as mean ± SD, *n*=3; ****, *P* < 0.0001; ***, *P* < 0.001; **, *P* < 0.01; *, *P* < 0.05; Student’s *t*-test.

**Supplementary Figure 5.** SATB1 ameliorates CAR-T cell exhaustion *in vitro.* (**A**) Schematic showing the CAR-T cell exhaustion model *in vitro* by co-incubation with Huh7 tumor cells. (**B**) PD-1 expression on CAR-T cells after co-incubation was detected by flow cytometry. (**C**) CTLA-4 expression on CAR-T cells after co-incubation was detected. (**D**) TIM3 expression on CAR-T cells after co-incubation was detected. (**E**) LAG-3 expression on CAR-T cells after co-incubation was detected. (**F**) PD-1 expression on CAR-T and SATB1-CAR-T cells after co-incubation was detected. Data were shown as mean ± SD, *n*=7; *, *P* < 0.05; Wilcoxon matched-pairs signed rank test. (**G**) CTLA-4 expression on CAR-T and SATB1-CAR-T cells after co-incubation was detected. Data were shown as mean ± SD, *n*=5; *, *P* < 0.05; paired two-tailed *t*-test. (**H**) TIM3 expression on CAR-T and SATB1-CAR-T cells after co-incubation was detected. Data were shown as mean ± SD, *n*=5; **, *P* < 0.01; paired two-tailed *t*-test. (**I**) LAG-3 expression on CAR-T and SATB1-CAR-T cells after co-incubation was detected. Data were shown as mean ± SD, *n*=5; **, *P* < 0.01; *, *P* < 0.05; paired two-tailed *t*-test.

**Supplementary Figure 6.** Overexpression of SATB1 ameliorated CAR-T cell exhaustion *in vivo*. (**A**) PD-1 expression on CD4^+^ and CD8^+^ CAR-T and SATB1-CAR-T cells isolated from tumors was detected. Data were shown as mean ± SD, *n*=4; ***, *P* < 0.001; **, *P* < 0.01; Student’s *t*-test. (**B**) CTLA-4 expression on CD4^+^ and CD8^+^ CAR-T and SATB1-CAR-T cells isolated from tumors was detected. Data were shown as mean ± SD, *n*=4; *, *P* < 0.05; Student’s *t*-test. (**C**) TIM3 expression on CD4^+^ and CD8^+^ CAR-T and SATB1-CAR-T cells isolated from tumors was detected. Data were shown as mean ± SD, *n*=4; **, *P* < 0.01; *, *P* < 0.05; Student’s *t*-test. (**D**) LAG-3 expression on CD4^+^ and CD8^+^ CAR-T and SATB1-CAR-T cells isolated from tumors was detected. Data were shown as mean ± SD, *n*=4; *, *P* < 0.05; Student’s *t*-test and Mann-Whitney test.
