# Supplementary figures and images for: Enhanced anti-liver tumor efficacy of chimeric antigen receptor-T cells via SATB1 modulation
Source: Cell Death Dis. 2025 Dec 10;17(1):93. doi: 10.1038/s41419-025-08307-3 (PMC12830387; doi:10.1038/s41419-025-08307-3)

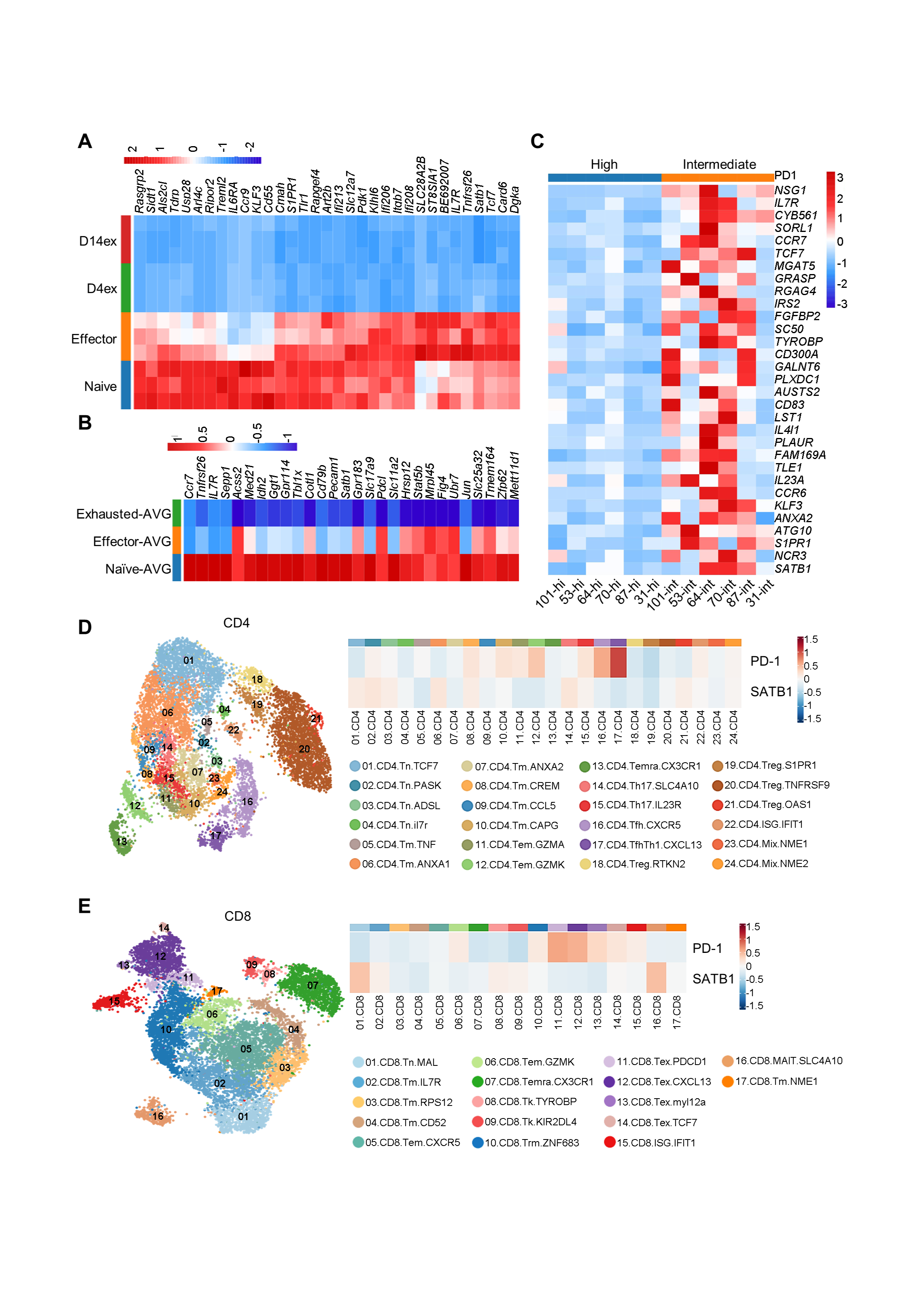

Supplement: Supplementary file 2 — Supplementary Figure S1 [file 41419_2025_8307_MOESM2_ESM.tif]

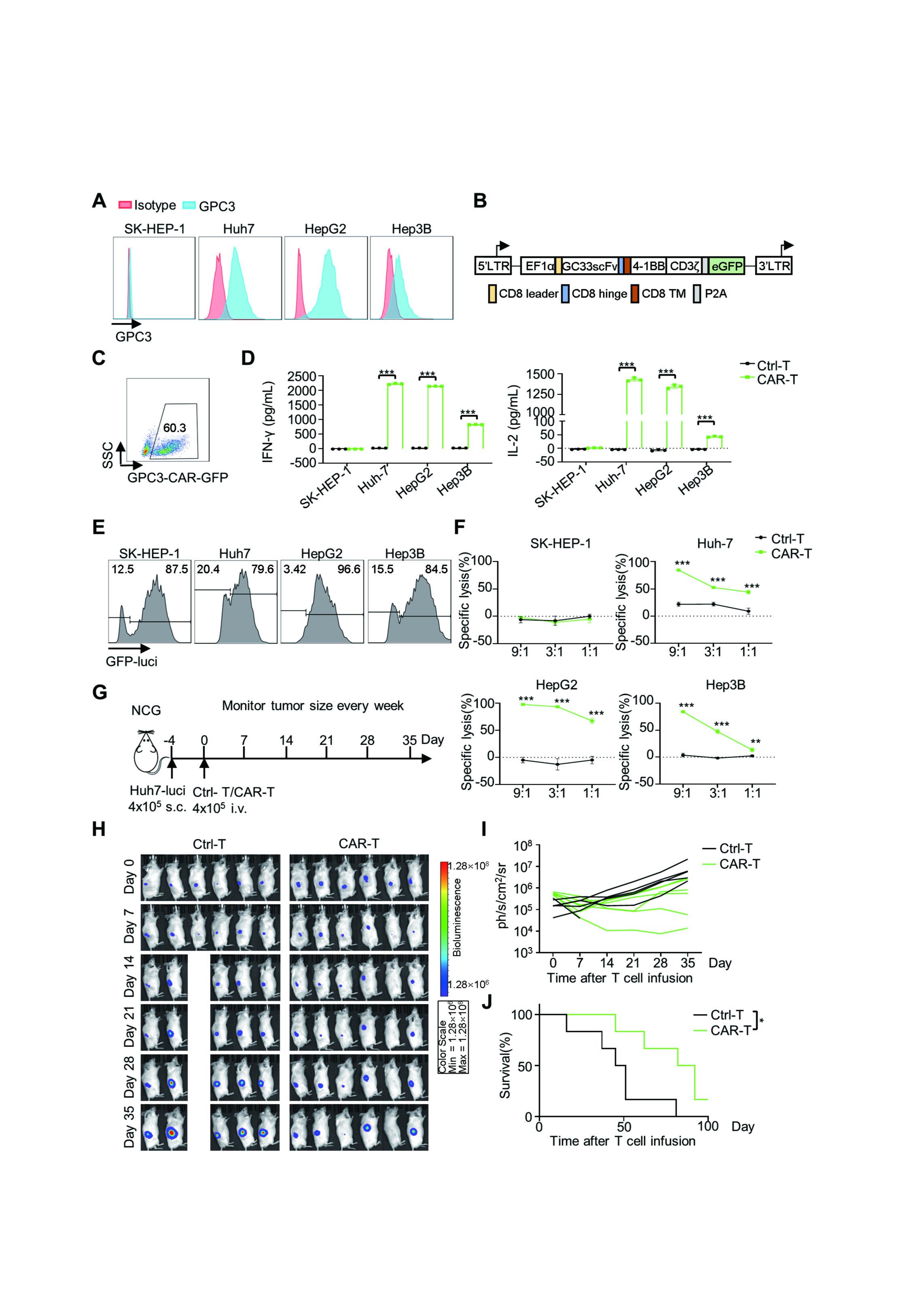

Supplement: Supplementary file 3 — Supplementary Figure S2 [file 41419_2025_8307_MOESM3_ESM.tif]

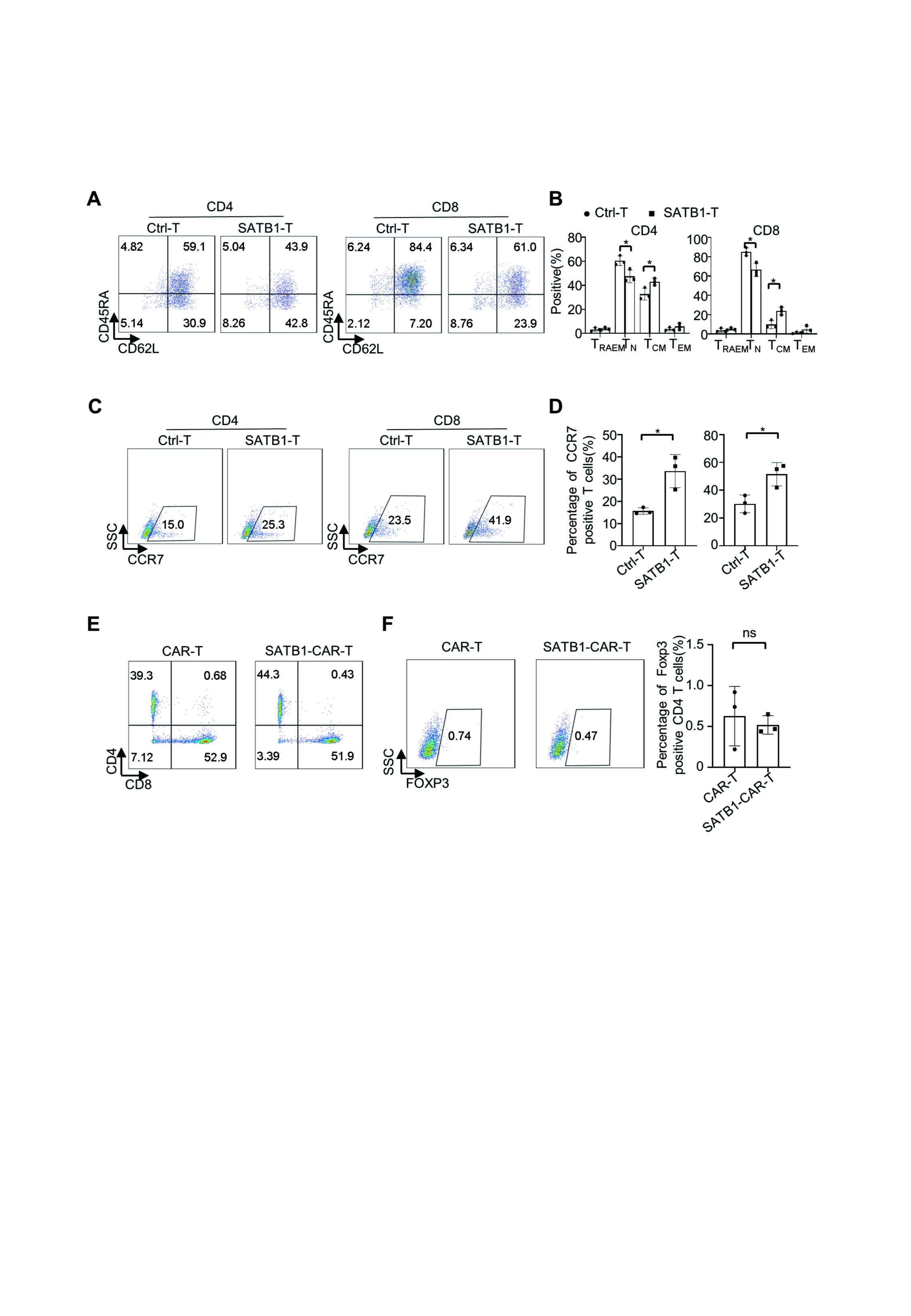

Supplement: Supplementary file 4 — Supplementary Figure S3 [file 41419_2025_8307_MOESM4_ESM.tif]

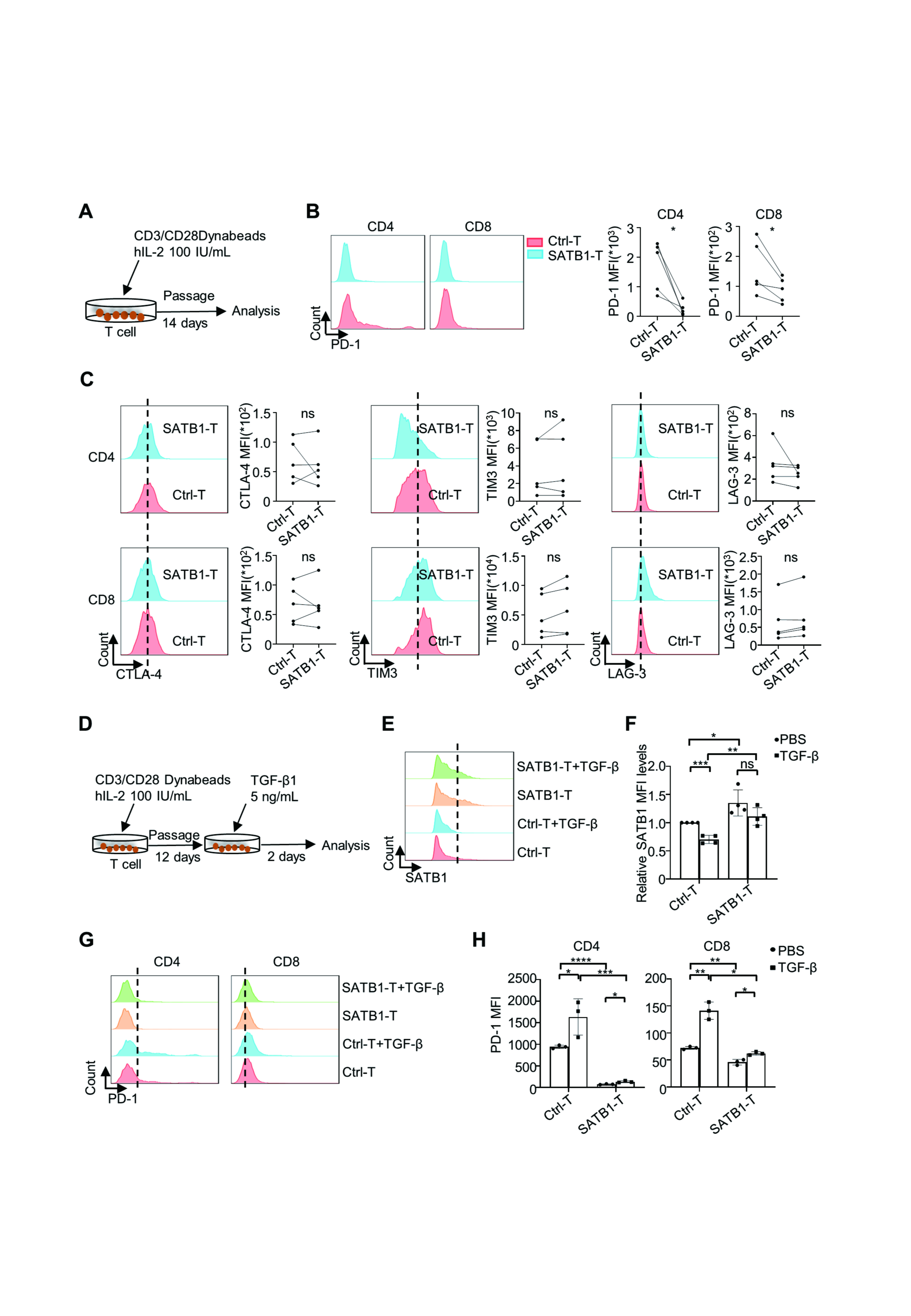

Supplement: Supplementary file 5 — Supplementary Figure S4 [file 41419_2025_8307_MOESM5_ESM.tif]

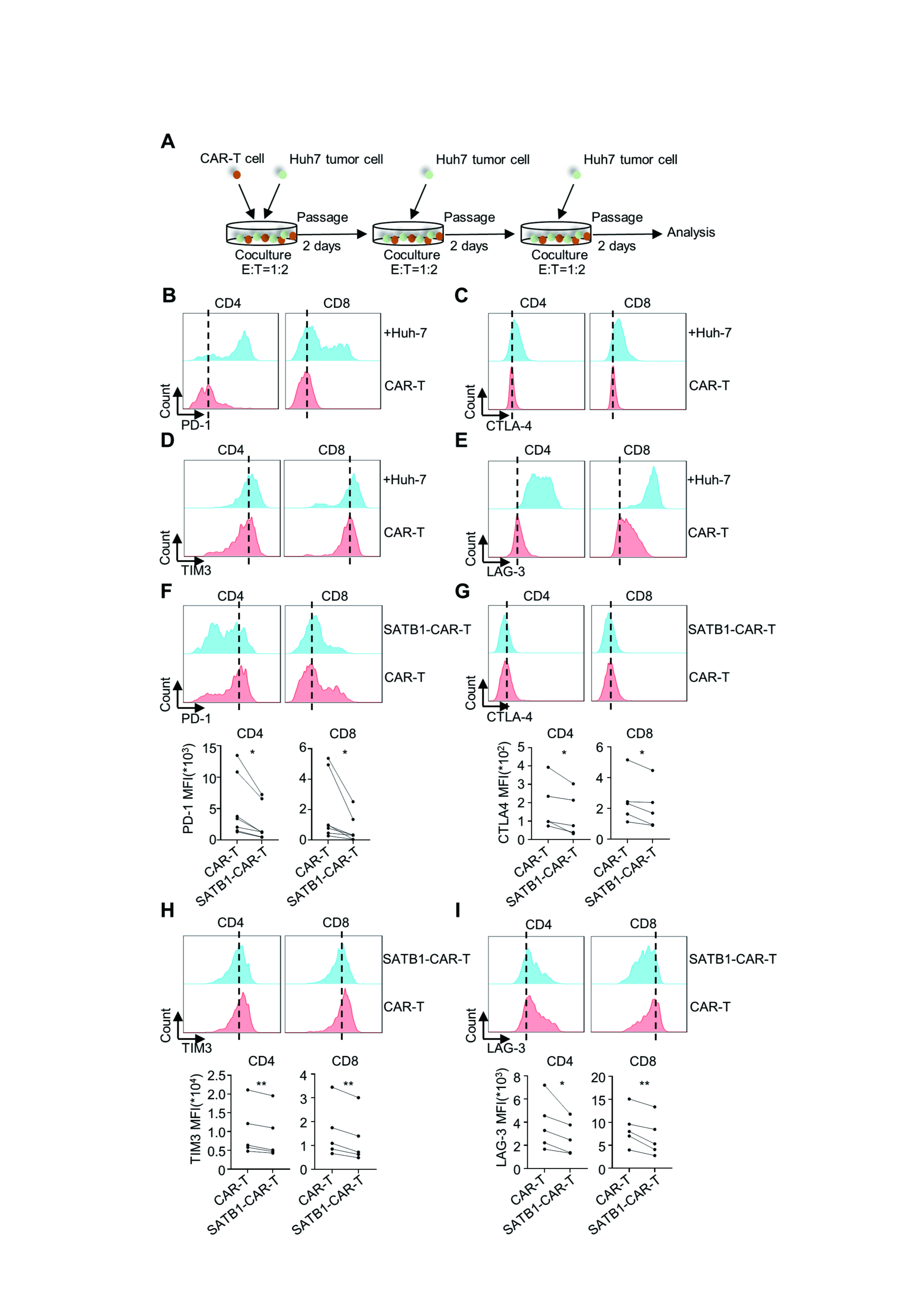

Supplement: Supplementary file 6 — Supplementary Figure S5 [file 41419_2025_8307_MOESM6_ESM.tif]

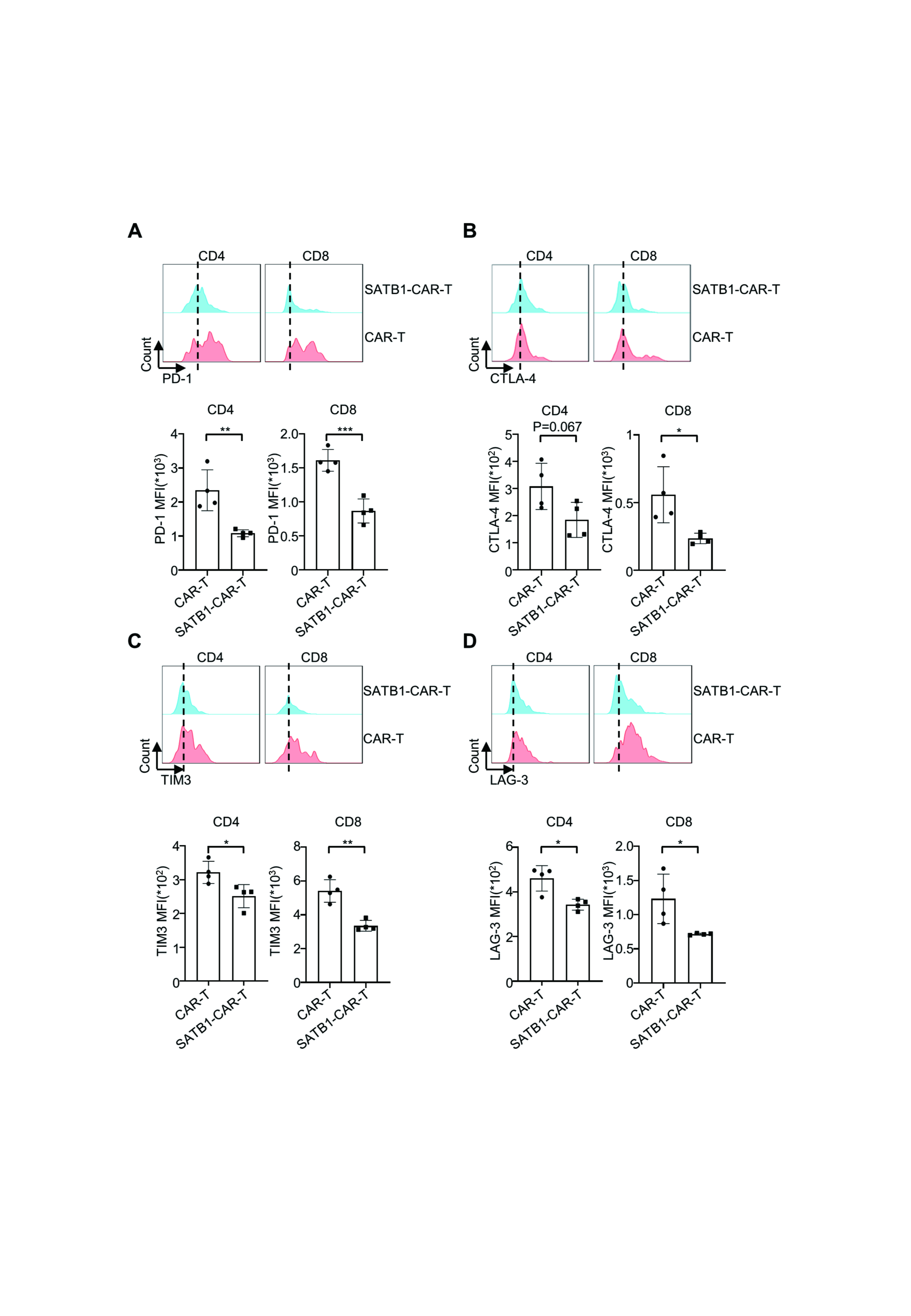

Supplement: Supplementary file 7 — Supplementary Figure S6 [file 41419_2025_8307_MOESM7_ESM.tif]
