# Supplementary material for: Enhanced anti-liver tumor efficacy of chimeric antigen receptor-T cells via SATB1 modulation
Source: Cell Death Dis. 2025 Dec 10;17(1):93. doi: 10.1038/s41419-025-08307-3 (PMC12830387; doi:10.1038/s41419-025-08307-3)
Supplement: Supplementary file 8 — Original Data Files [file 41419_2025_8307_MOESM8_ESM.pptx]

## Slide 1
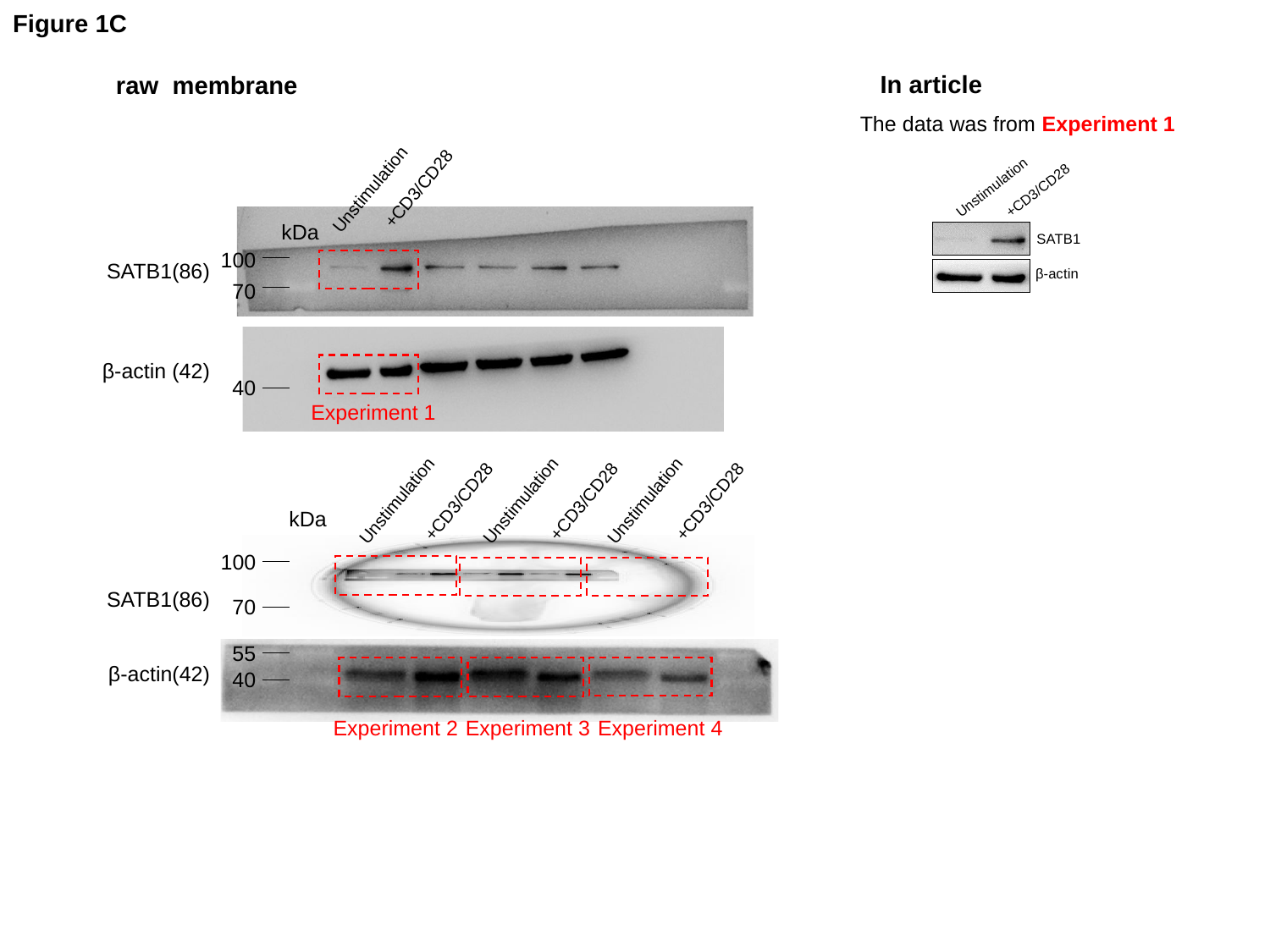

Figure 1C
In article
 raw membrane
The data was from Experiment 1
Unstimulation
+CD3/CD28
SATB1
β-actin
+CD3/CD28
Unstimulation
kDa
100
SATB1(86)
70
β-actin (42)
40
Experiment 1
Unstimulation
Unstimulation
Unstimulation
+CD3/CD28
+CD3/CD28
+CD3/CD28
kDa
100
SATB1(86)
70
55
β-actin(42)
40
Experiment 2
Experiment 3
Experiment 4

## Slide 2
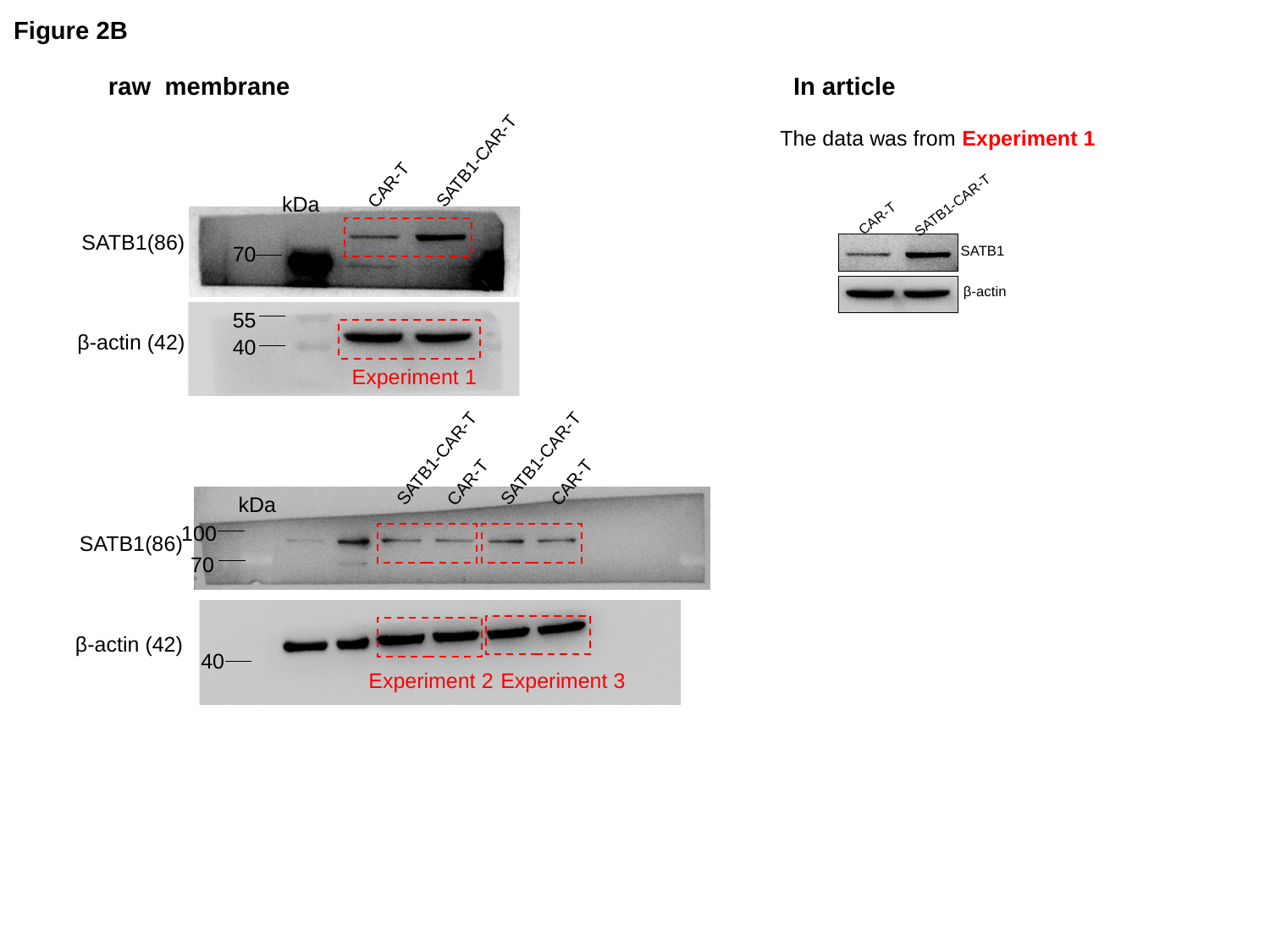

Figure 2B
 raw membrane
In article
The data was from Experiment 1
SATB1-CAR-T
CAR-T
kDa
SATB1-CAR-T
CAR-T
SATB1
β-actin
SATB1(86)
70
55
β-actin (42)
40
Experiment 1
SATB1-CAR-T
SATB1-CAR-T
CAR-T
CAR-T
kDa
100
SATB1(86)
70
β-actin (42)
40
Experiment 2
Experiment 3
